# The impact of COVID-19 on the occupational health of oncologists: a descriptive analysis of occupational safety, perceived burnout and social support among practicing oncologists in Alexandria, Egypt

**DOI:** 10.3332/ecancer.2021.1273

**Published:** 2021-07-28

**Authors:** Abeid M A Omar, Marwa M Ramadan, Yomna Khamis, Abdelsalam A Ismail

**Affiliations:** 1 Department of Clinical Oncology and Nuclear Medicine (ACOD), Faculty of Medicine, Alexandria University, Champillion Street – Khartoum Square, 21131 Alexandria, Egypt; 2 Department of Community Medicine, Faculty of Medicine, Alexandria University, Champillion Street – Khartoum Square, 21131 Alexandria, Egypt; a https://orcid.org/0000-0003-4081-8547; b https://orcid.org/0000-0003-4953-7346; c https://orcid.org/0000-0001-8531-0849

**Keywords:** COVID-19, burnout, oncologists, isolation, occupational health

## Abstract

**Background:**

Healthcare workers, including oncologists, face a higher potential risk of contracting coronavirus disease 2019 (COVID-19) while managing patients. Moreover, the uncertainty that came with COVID-19 and its associated social stigma may worsen what was already a crisis (burnout) among oncologists. Data are scarce on the impact of COVID-19 on the occupational health and safety of oncologists in low and middle-income countries.

**Methods:**

We conducted a cross-sectional survey in February 2021 to evaluate the impact of COVID-19 on practicing oncologists in Alexandria governorate, Egypt. An anonymised self-reporting questionnaire was electronically distributed to 88 participants to collect information on occupational safety at work, the prevalence of COVID-19 among respondents and the impact of COVID-19 on their wellbeing, including perceived burnout and family support.

**Results:**

Out of the 88 contacted oncologists, 75% completed the survey. The mean age of participants was 34.79 years (SD ± 10.42), of which 45% were residents, 36% were specialists and 18% were consultants. Most of the oncologists (58% of 66) felt they were not adequately protected against COVID-19. The majority (78% of 66) have managed COVID-19 infected cancer patients, and 76% (out of 66) had experienced COVID-19 like symptoms. A third (*n* = 21) of the respondents were confirmed COVID-19 infected: 62% of the latter thought they were infected at the workplace, either by a patient or a colleague. The majority of the oncologists (78%) perceived being more overwhelmed or burned out than in the pre-COVID-19 era. Nearly half of the participants (48%) reported their family members and friends had reduced contact with them despite being COVID-19 negative, in fear of being infected. The burnout was significantly higher in those lacking family support than those who had, 52% versus 28% respectively (*p* = 0.038).

**Conclusions:**

One-third of practicing oncologists were diagnosed with COVID-19, and most thought they were infected at the hospital. Occupational safety measures, including mental health programs, need to be improved with special emphasis on the role of family support in mitigating perceived burnout among practicing oncologists.

## Introduction

According to World Health Organisation, as of March 2021, more than 115,000,000 people were infected with coronavirus disease 2019 (COVID-19) worldwide [1]. Healthcare workers (HCW) face a higher potential risk of contracting COVID-19 and even death [2, 3]. This is compounded by the shortage of personal protective equipment (PPE) worldwide [4]. By August 2020, more than 300,000 HCW workers had been infected globally, of which a third were doctors [5], worsening the shortage of physicians.

It is well-known that physicians experience burnout, even among the most resilient [6]. Among the physicians, oncologists have one of the highest burnout rates [7]. The uncertainty surrounding COVID-19 worsened what was already a crisis (burnout) among the oncologists [7, 8]. According to the Medscape Oncologist Lifestyle, Happiness & Burnout Report 2021, there is a 15% increase in the number of oncologists experiencing burnout since the beginning of COVID-19 [9].

Historically, human immunodeficiency virus, hepatitis C virus and tuberculosis-infected people face social stigma and discrimination in the community [10]. COVID-19, being an infectious disease, is not an exception to this [11]. Increasing evidence shows that HCW, COVID-19 patients and survivors face stigma from the community as they are considered as a source of infection [12].

COVID-19 has also contributed to psychological disturbances among oncologists. Some physicians are experiencing anxiety and depression and have resolved to use psychostimulants, tobacco or even alcohol [13].

While most of the previous studies assessing the impact of COVID-19 on oncologists were done in the western world, little has been reported in the low and middle-income countries (LMICs). In light of this, we conducted this study to assess the impact of COVID-19 on the occupational health of oncologists in Alexandria, Egypt, including occupational safety at work, their prevalence of COVID-19 and the impact of COVID-19 on perceived burnout and family support.

## Methodology

We conducted a voluntary cross-sectional survey among oncologists practicing in public and the private sectors of Alexandria governorate, Egypt. In the first week of February 2021, an anonymised electronic google form (Appendix 1) was distributed by email and social media to 88 oncologists. These tools were selected for their familiarity in our set-up, even among the senior oncologists, and the associated risk of in-person data collection during the pandemic. The questionnaire was distributed in English language and included information on the demographic characteristics, occupational safety at work, the prevalence of COVID-19 among respondents, the impact of COVID-19 on their wellbeing, including perceived burnout and the attitude of their families and friends towards them. Burnout as a syndrome was not assessed as a part of the present analysis as the condition was already established in pre-pandemic studies [14], but rather we were interested in assessing the work environment and social support amid the pandemic and asked oncologists directly whether they are feeling more overwhelmed or burned out compared to the pre-pandemic era. Data on gender and the specific type of health center were also not collected to ensure the anonymity of respondents as some centers had one or two practicing oncologists. Descriptive analysis was used to characterise the participants and was reported in numbers and percentages. Pearson Chi-square and two-sample *t*-test were used to compare the different variables. *p*-value < 0.05 was considered to be significant. Data were analysed by the STATA software version 16.

This survey was approved by the ethical committee of Alexandria University, Egypt, as a part of the ongoing work assessing the impact of COVID-19 on cancer care.

## Results

### Sociodemographic characteristics

**Figure 1.**
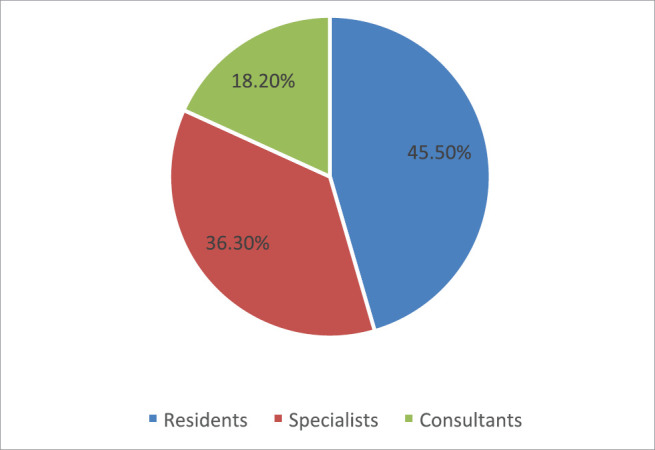
The distribution of the respondents according to their ranks.

Out of the 88 contacted oncologists, 75% (*n* = 66) completed the survey, whereby nearly half were residents (45.5%), 36.3% were specialists and 18.2% were consultants (Figure 1). The mean age of the participants was 34.78 years (SD ± 10.42).

### Safety at the workplace and attitude towards COVID-19

Table 1 summarises the results of this study. Regarding protection against COVID-19 at the workplace, most physicians felt they are not well protected (58%). In contrast, very few feel they are well protected (3%). Out of the 66 respondents, only a few oncologists (21%) never contacted a COVID-19 cancer patient. Noteworthy, only 12% of the participating physicians were comfortable treating infected cancer patients. Nearly all physicians (98%) have worked with a previously COVID-19 infected colleague. More than half (55%) are willing to work with colleagues regardless of the infection status, and 36% are comfortable working with colleagues who are currently not infected. However, 9% responded they would only work with a colleague who they are sure never had a history of COVID-19.

### The impact of COVID-19 on wellbeing and social life (stigma)

More than three-quarters of the respondents (79%) reported are experiencing more burnout than before the COVID-19 pandemic. Nearly half (48%) of the participants indicated their family members and friends had reduced contact with them as they fear they could be infected. Contrary to this, a quarter (24%) reported that family and friends are now more supportive.

### Factors associated with burnout

**Figure 2.**
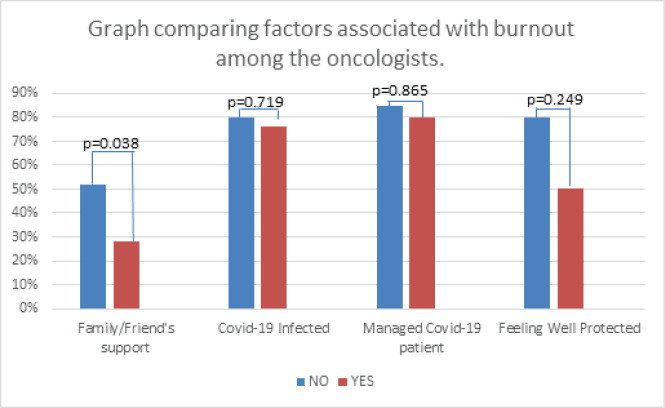
Factors potentially associated with burnout among the oncologists.

The physicians lacking family and/or friends’ support experienced significantly more burnout than those who had support, 52% versus 28% respectively; *p* = 0.038. Those diagnosed with COVID-19 had similar burnout compared to those uninfected, 76% versus 80%, respectively; *p* = 0.719. Similarly, the burnout level was comparable among the physicians who never treated COVID-19 patients to those who have managed at least once and those treated more than once, 85% versus 80% versus 78%, respectively; *p* = 0.865. Furthermore, oncologists who felt are well protected had the same burnout level as those who felt they were not well protected, 50% versus 80%, respectively; *p* = 0.249. Finally, there was no difference in burnout level, neither by ranks (90% in consultants versus 74% in residents versus 83% in specialists; *p* = 0.487) nor age. Figure 2 compares the burnout according to the factors mentioned earlier.

### Infection and coping with COVID-19

**Figure 3.**
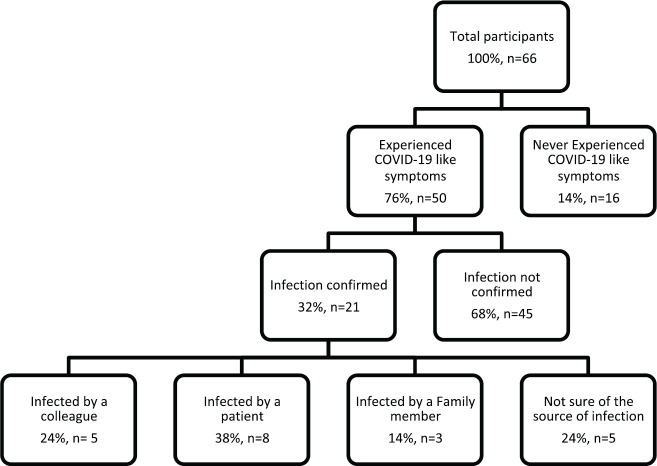
Frequency of COVID-19 symptoms and presumed source of infection.

**Figure 4.**
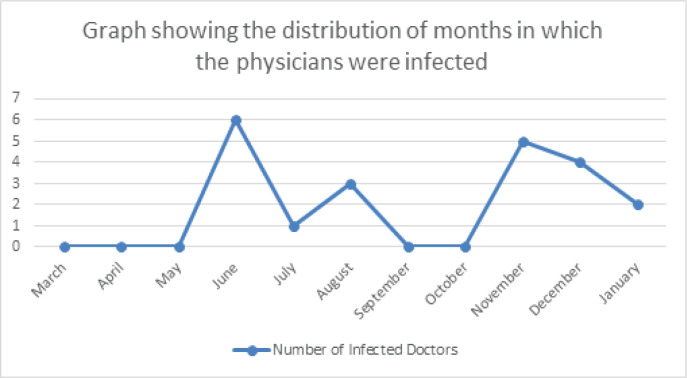
Distribution of confirmed COVID-19 cases by month.

**Figure 5.**
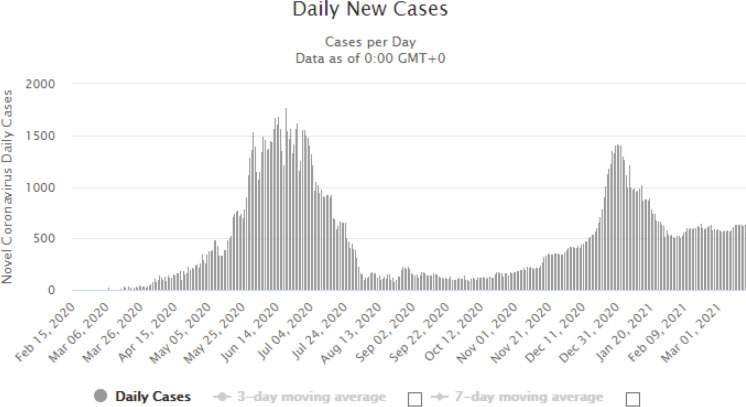
Daily COVID-19 cases in Egypt. Source: https://www.worldometers.info/coronavirus/country/egypt/.

More than three-quarters of the physicians (76%) have experienced COVID-19 like symptoms such as fever, cough and loss of sense of smell, whereas the remaining had never experienced such symptoms. Nevertheless, only a third of the physicians (*n* = 21) were confirmed to be COVID-19 positive. Among the infected physicians, most thought they were infected by the patients (38%), whereas a quarter by the colleagues, and a minority by their families (14%), while 24% could not identify their infection source, as illustrated in Figure 3. The infection period varied among the physicians; most were infected in June (29%) and November (19%), which coincides with the national peak incidence of COVID-19, as illustrated in Figures 4 and 5 [15].

In coping with the COVID-19, two-thirds of the infected physicians received moral support from their colleagues and the family. At the same time, the remaining third had only the family to support them.

### Relationship of COVID-19 with working conditions, rank and age

Out of the 21 infected physicians: 27% were consultants, 39% were residents and 30% were specialists. There was no relationship between the chance of being infected and the physician’s rank (*p* = 0.632). The physician’s age did not increase the likelihood of being diagnosed with COVID-19 (*p* = 0.288). Furthermore, those who felt they were well protected to carry out their daily activities had an equal chance of being infected (50%), similarly to those who thought they were not well protected (31%) or neutral (35%); *p* = 0.823.

## Discussion

Our findings suggest that oncologists do not feel well protected and not comfortable performing their duties amid the COVID-19 pandemic. Moreover, most doctors have worked with an infected colleague or treated a COVID-19 patient. Most physicians had COVID-19 like signs, with 32% being confirmed positive. There was no association between the chance of being infected and the oncologist’s age, rank or feeling well protected. Some oncologists had experienced some stigma at home. They reported that their family members and friends had reduced contact with them in fear of being infected. Although most oncologists had worked with a once infected colleague, a few reported would not work with a once infected colleague.

The emergency of the COVID-19 has created fear and stigma among the public. In this survey, nearly half of the respondents experienced some degree of stigma by their family members and friends in fear of being infected. Although almost all physicians had worked with an infected colleague, some of the respondents, albeit very few, reported they were unwilling to work with a once-infected colleague. Similarly, in a Turkish study assessing stigma among HCW, they found that HCW with COVID-19 symptoms or working with COVID-19 patients experienced more stigma than those without symptoms or those not working with COVID-19 patients [16]. Likewise, in another study conducted in North America, a third of non-HCW reported avoiding HCW; they even wanted them to be isolated and not to stay with their families to avoid infecting the community [17]. As corona is taking longer to be contained globally, these data indicate an urgent need to educate the general public and professionals’ attitudes towards infected colleagues, relatives or health professionals attending to the infected patients.

This work showed that most oncologists in Alexandria had COVID-19 exposure, similar to the study by Kim *et al* [18] in which 83% of HCW reported COVID-19 exposure. Moreover, we found the majority (78%) of the physicians had experienced COVID-19 signs, like fever, cough and loss of smell, which is similar to that reported in the Korean study where 70% of the respondents had experienced a loss of sense of smell and test before being diagnosed with COVID-19 [19]. Nevertheless, only 32% of our participants were confirmed having COVID-19, which corresponds to a study conducted in Paris, whereby out of the symptomatic HCW, only 28% were confirmed to be COVID-19 positive [20]. This could be due to the lack of readily available COVID-19 testing in our setup. Therefore, more testing is needed in symptomatic HCW to rule out infection, as sharing work environment is associated with an increased risk for COVID-19 [21].

COVID-19 is a very contagious disease and poses an occupational hazard without proper protection at the workplace [22, 23]. Compared to other professionals, HCW can trace the origin of their infection more reliably [24]. Nearly two-thirds of the infected oncologists reported were infected at the hospital either by patients or colleagues. It is not surprising as most physicians had managed infected patients and felt well not protected at their workplace. In the Italian national survey, only a fifth of the participants felt they had enough and good quality PPE, and it is reflected among the infected HCW, whereby 89% reported they were infected at the workplace [25]. In the USA, 28% of the HCW reported to had been infected at households [26] similar to this study where 24% attributed the family to be their infection source. To ensure occupational safety, the hospital administration should provide HCW with adequate and quality PPE and speed the facilitation and prioritisation of vaccines during the vaccination process. At the writing of this paper, the government has started rolling out vaccines.

Young age, female gender, working with infected patients and lacking or having inadequate PPE have been identified as risk factors of COVID-19 infection among HCW [27–29]. However, in this study, there was a similar chance of being infected no matter the rank, age, having treated COVID-19 patients or whether one had the feeling of well-protected at the workplace, and these results resonate well with what was reported by Felice *et al* [25].

Burnout is common among physicians, and oncologists are not an exception due to their work nature [30]. A study conducted in Argentina, a LMIC, during the third wave of COVID-19 pandemic found the burnout among the medical oncologists to vary between 14.9 and 47.9 based on different burnout scales they used [31]. In Egypt, before the COVID-19 pandemic, a single institution study showed oncologists were suffering from burnout: 72% had emotional exhaustion, 49% depersonalization, and 38% personal accomplishment [32]. After the beginning of the COVID-19 pandemic, a multi-center study reported 32% of the Egyptian oncologists having burnout syndrome [33]. The uncertainty brought by COVID-19 has worsened the situation and could negatively impact the physicians’ performances [34]. In this study, we found that 79% of the participants had an increased burnout rate than the pre-pandemic. This is consistent with the Medscape Oncologist Lifestyle, Happiness & Burnout Report 2021, where they found 32% of the oncologists had burnout syndrome; 85% had started before COVID-19 compared to 15%, which started in the COVID-19 period. Similarly, the recently published European Society for Medical Oncology Task Force reported that 38% of the oncologists experienced burnout syndrome, and two-thirds could not perform their work well compared to before COVID-19 time [35]. Hence, actions are required to counteract the negative consequences of burnout syndrome.

In the predictors of burnout, the lack of family and/or friend’s support during the COVID-19 pandemic was significantly associated with burnout. However, we found that the physician’s rank was not associated with burnout, similarly reported in the USA, where they found the junior and senior oncologists had comparable burnout syndrome [36]. In this study, the physicians who felt are well protected had comparable burnout syndrome with those who perceived they were not well protected at work. Moreover, we found that burnout was similar among the infected and non-infected colleagues. To the authors’ knowledge, this work is among the few studies comparing burnout among the infected and non-infected HCW. Since it is well-known that oncologists have been experiencing high burnout even before the pandemic, this could explain why rank, age, and most other factors were not associated with burnout among the oncologists in this study.

## Conclusions

This study has exposed COVID-19 as an occupational hazard. There is an unmet need for proper protection at the workplace as most participants felt they were not well protected at the workplace, and some ended up being confirmed with COVID-19. Proper protection will prevent burdening the already overwhelmed oncologists with more workload due to sick colleagues. Moreover, more screening and COVID-19 testing should be performed on oncologists and patients presenting with COVID-19 like symptoms to prevent cross infection at the workplace. Nevertheless, we commend the government for launching the rollout of the COVID-19 vaccine. However, they should speed up the vaccination process not only for HCW but also the cancer patients. In addition, the ministry of health should sensitise the public against discriminating against COVID-19 patients and HCW. Furthermore, mental health support should be provided to HCW as burnout was highly prevalent among the participants and had manifested even before the COVID-19 era. Finally, we highly recommend a national survey to be carried out with a larger sample size, covering the oncologists and other HCW using the established burnout syndrome scales to better understand and compare burnout syndrome among different HCW in Egypt.

## Limitations

This study had a small sample size as it involved only oncologists working in one region of Egypt, Alexandria. Yet, this region is crucial as it contributes to the national and International Agency for Research on Cancer cancer registry. Nevertheless, these findings need to be interpreted cautiously. Different scales have been developed to assess burnout syndrome; in the present study, participants were asked directly whether they perceive more burnout compared to the pre-pandemic era. Thus, these results need to be cautiously interpreted as burnout may refer only to the perception of being ‘overwhelmed’ or ‘exhausted’. Despite these limitations, this study is important as very few studies have assessed the impact of COVID-19 among oncologists in LMIC countries, hence making a significant contribution in understanding the magnitude in this set-up.

## Conflicts of interest

All of the authors declare that they have no conflicts of interest

## Funding

This work did not receive any funding.

## Appendix 1. Questionnaire.

**Table 1.** Summary of the questions asked and the results.

Question	Options	Results	
		n	%
Have you ever had to treat a cancer COVID-19 patient?	Never	14	21
	Once	6	9
	More than one time	46	70
How comfortable are you managing cancer patients during the COVID-19 pandemic?	Comfortable	37	56
	Not comfortable	21	32
	Neutral	8	12
Do you feel well protected to attend daily at the hospital?	Not protected at all	38	58
	Very protected	2	3
	Neutral	26	39
Do you feel more burned out than before the COVID-19?	No, (same as before COVID-19)	13	21
	Yes, (more overwhelmed than before COVID-19)	50	79
Have you worked with a colleague who was diagnosed with a COVID-19?	Yes	61	98
	No	1	2
You feel comfortable working with a colleague, only if?	I am sure they have no history of COVID-19 infection	6	9
	Assumes themselves are free from COVID-19 infection	24	36
	No difference	36	55
As a physician (and COVID-19 free), did your family and friends reduce physical contact with you since COVID-19 started? (because they are afraid you could infect them)	No, the same level of contact	18	27
	Yes, they are afraid I could infect them	32	49
	They are now more supportive	16	24
Since the beginning of COVID-19, have you ever had COVID-19 like symptoms (fever, cough, loss of sense of smell etc)?	Yes	50	76
	Never	16	24
Have you been diagnosed with COVID-19?	Yes	21	33
	No	43	67
Did you get moral support from colleagues/or family?	Yes, Only by family	7	33
	Yes, both family and colleagues	13	62
	I prefer Not to say	1	5

## References

[1] WHO coronavirus (COVID-19) dashboard. https://covid19.who.int/table.

[2] Bandyopadhyay S, Baticulon RE, Kadhum M (2020). Infection and mortality of healthcare workers worldwide from COVID-19: a systematic review. BMJ Glob Health.

[3] Ing EB, Xu QA, Salimi A (2020). Physician deaths from corona virus (COVID-19) disease. Occup Med (Lond).

[4] Jain U (2020). Risk of COVID-19 due to shortage of personal protective equipment. Cureus.

[5] Erdem H, Lucey DR (2021). Healthcare worker infections and deaths due to COVID-19: a survey from 37 nations and a call for WHO to post national data on their website. Int J Infect Dis.

[6] West CP, Dyrbye LN, Sinsky C (2020). Resilience and Burnout among physicians and the general US working population. JAMA Netw Open.

[7] Eelen S, Bauwens S, Baillon C (2014). The prevalence of burnout among oncology professionals: oncologists are at risk of developing burnout. Psychooncology.

[8] Soltan MR, Soliman SS, Al-Hassanin SA (2020). Burnout and work stress among medical oncologists: Egyptian multi-centric study. Middle East Curr Psychiatry.

[9] Medscape Oncologist Lifestyle (2021). Happiness & Burnout Report.

[10] Baldassarre A, Giorgi G, Alessio F (2020). Stigma and discrimination (Sad) at the time of the sars-cov-2 pandemic. Int J Environ Res Public Health.

[11] Chopra KK, Arora VK (2020). COVID-19 and social stigma: role of scientific community. Indian J Tuberc.

[12] Bagcchi S (2020). Stigma during the COVID-19 pandemic. Lancet Infect Dis.

[13] Hilmi M, Boilève A, Ducousso A (2020). Professional and psychological impacts of the COVID-19 pandemic on oncology residents: a national survey. JCO Glob Oncol.

[14] AbdAllah M, El-Hawy L (2019). Burnout and health related quality of life among resident physicians in Zagazig University Hospitals. Egypt J Occup Med.

[15] Coronavirus Cases in Egypt. https://www.worldometers.info/coronavirus/country/egypt/.

[16] Teksin G, Uluyol OB, Onur OS (2020). Stigma-related factors and their effects on healthcare workers during COVID-19 pandemics in Turkey: a multi-center study. Sisli Etfal Hastan Tip Bul.

[17] Taylor S, Landry CA, Rachor GS (2020). Fear and avoidance of healthcare workers: an important, under-recognized form of stigmatization during the COVID-19 pandemic. J Anxiety Disord.

[18] Kim R, Nachman S, Fernandes R (2020). Comparison of COVID-19 infections among healthcare workers and non-healthcare workers. PLoS One.

[19] Villarreal IM, Morato M, Martínez-RuizCoello M (2020). Olfactory and taste disorders in healthcare workers with COVID-19 infection. Eur Arch Otorhinolaryngol.

[20] Lee S, Persson P, Mathews RD (2020). Comparing dynamics and determinants of SARS-CoV-2 transmissions among health care workers of adult and pediatric settings in central Paris. Clin Infect Dis.

[21] Garzaro G, Clari M, Ciocan C (2020). COVID-19 infection and diffusion among the healthcare workforce in a large university-hospital in Northwest Italy. Med Lav.

[22] Zunyou W, McGoogan JM (2020). Characteristics of and important lessons from the coronavirus disease 2019 (COVID-19) outbreak in China. JAMA.

[23] Huang Z, Zhuang D, Xiong B (2020). Occupational exposure to SARS-CoV-2 in burns treatment during the COVID-19 epidemic: specific diagnosis and treatment protocol. Biomed Pharmacother.

[24] Id FL, Wei C, Id YH (2020). Work-related COVID-19 transmission in six Asian countries/areas: a follow-up study. PLoS One.

[25] Felice C, Luca G, Tanna D (2020). Impact of COVID-19 outbreak on healthcare workers in Italy: results from a national e-survey. J Community Health.

[26] Burrer SL, Perio MA, Hughes MM (2020). Characteristics of health care personnel with COVID-19. MMWR Morb Mortal Wkly Rep.

[27] Çelebi G, Pişkin N (2020). Specific risk factors for SARS-CoV-2 transmission among health care workers in a university hospital. Am J Infect Control.

[28] Al Maskari Z, Al Blushi A, Khamis F (2021). Characteristics of healthcare workers infected with COVID-19: a cross-sectional observational study. Int J Infect Dis.

[29] Wei J, Liu Z, Fan Z (2020). Epidemiology of and risk factors for COVID-19 infection among health care workers: a multi-centre comparative study. Int J Environ Res Public Health.

[30] Murali K, Banerjee S (2018). Burnout in oncologists is a serious issue: what can we do about it?. Cancer Treat Rev.

[31] Andres G, Gonzalo P, Federico W (2021). Burnout syndrome in medical oncologists during the COVID-19 pandemic: Argentinian national survey. ecancer.

[32] Ghali R, Boulos D, Alorabi M (2019). Cross-sectional study of Burnout among a group of Egyptian oncologists at Ain Shams University. Res Oncol.

[33] Soltan MR, Soliman SS, Al-Hassanin SA (2020). Burnout and work stress among medical oncologists: Egyptian multi-centric study. Middle East Curr Psychiatry.

[34] Hlubocky FJ, Symington BE, McFarland DC (2021). Impact of the COVID-19 pandemic on oncologist Burnout, emotional well-being, and moral distress: considerations for the cancer organization’s response for readiness, mitigation, and resilience. JCO Oncol Pract.

[35] Banerjee S, Jonathan Lim KH, Murali K (2021). The impact of COVID-19 on oncology professionals: results of the ESMO resilience task force survey collaboration. ESMO Open.

[36] Shanafelt TD, Raymond M, Horn L (2014). Oncology fellows' career plans, expectations, and wellbeing: do fellows know what they are getting into?. J Clin Oncol.

